# Nuances of an In-Between Space of Learning Through Auditory Approaches in Early Piano Instruction

**DOI:** 10.3390/bs14121128

**Published:** 2024-11-25

**Authors:** Samuel E. Pang, Rebecca Y. P. Kan

**Affiliations:** Nanyang Academy of Fine Arts, University of the Arts Singapore, Singapore 189655, Singapore; ypkan@nafa.edu.sg

**Keywords:** aural development, piano pedagogy, early piano instruction, applied studio teaching, in-between space of learning

## Abstract

Musical experiences in early piano instruction tend to be led by visual-based methods, limiting opportunities to develop aural abilities for children to understand music. This study examines the exploratory behaviour of music listening through auditory approaches that support visual-based methods to foster musical comprehension. Drawing from case studies of young music learners between the ages of 7 and 8, qualitative data were collected through lesson observations, interviews, game-based assessments, and performance evaluations of a prepared piece. Positive instances of recall, calibration, association, and empowerment indicated how participants perceived and strengthened the association of heard sounds. The findings further highlight the demanding cognitive ability needed to process visual elements in method books and how auditory approaches can relieve the attention to visual score-reading that enables students to better tune in to the coordination of hands with music. This discussion therefore opens the possibility for exploring how we may uncover nuanced differences in learning when we design teaching methods that straddle both auditory and visual approaches.

## 1. Introduction

In Singapore, piano method books are popular pedagogical materials for practitioners to teach beginner-level students. The variety of books employed are either the teacher’s personal preferences or dictated by music schools. Typical examples include the *Piano Adventures Series* by Nancy and Randall Faber [1] and *Piano Lessons Made Easy Series* by Lina Ng [2]. These books often incorporate essential fundamentals of understanding the instrument, technique, music notation, music theory, scales and arpeggios, sight reading, and aural awareness to enable beginners to reproduce music on the keyboard. Their clever design and colourful graphical illustrations often attract and capture the attention of the young.

Yet, we find that such musical experiences tend to be led by visual-based methods, limiting opportunities to develop aural abilities for children to understand music [3]. There is still insufficient knowledge about how listening behaviours take place in early piano instruction. While auditory approaches do exist [4], there are still no empirical studies on the switching of both visual and auditory teaching approaches in early piano instruction. This has led to a dearth of information about toggling from visual to auditory practices and vice versa to develop musical comprehension.

This article identifies an in-between-ness when auditory approaches are integrated to support visual approaches. This paper illustrates an addressable gap in the design of pedagogical approaches in early piano instruction. The literature review contends that early piano pedagogy can be productively conceptualised by exploring both aural and visual approaches that are in creative tension. We describe the design of teaching methods that straddle both auditory and visual approaches in three case studies. Representing pedagogy in such a way draws out the nuances of an in-between space of learning [5]. It also creates possibilities for us to consider how teachers may better support musical development by providing students with the capabilities to simultaneously develop both auditory and visual literacies through music.

## 2. Tensions in Pedagogical Orientations of Early Piano Instruction

In recollections of creative strategies for music teaching and learning, McPherson and Gabrielsson [6] recounted traditions of learning an instrument and its musical knowledge through the master–apprentice approach, where rote learning by imitating and modelling the master’s playing is prevalent. Modern method books, however, have placed a bias on visual-based approaches to “associate fingers with notation rather than fingers with sound, perpetuate the mathematical relationships of proportional note values, emphasise note naming and theoretical concepts more than perceptual understanding, and separate technical skill from the process of learning to play actual music” [6] (p. 101). For example, the scale of C is taught through visual illustrations with fingerings, letter names, and hand placement. In another instance, high and low registers are taught through visual associations in pre-staff notation instead of allowing the learners to experience the differences in register aurally. Likewise, Bunting [7], Williams [8], and Arshinova [9] concurred that in today’s early piano instruction, visual musical notation literacy is prioritised.

The reproduction of music appears to be at the core of early piano instruction, and piano method books efficiently encompass the essential and fundamental skills needed to manipulate the instrument. Method books present various forms of graphical illustration to concepts such as the recognition and placing of hand and fingers, finger numbers, letter names, keyboard geography, keyboard range, black and white keys, and directions [3]. Well-designed materials would typically include note reading right from the first lesson [10,11], as well as basic music theory [12] and the technical aspects of the piano, often with colourful graphical illustrations that attract and capture the child’s attention. It is therefore not peculiar as to why piano teachers are drawn to employ these materials within their teaching practices [12,13]. However, this promotes a theory-driven understanding of music to achieve the goal of reproducing music.

This is not to say that there are no auditory-based approaches in early piano instruction. Rather, such experiences are usually marginalised. For instance, in *Music Little Mozarts: Music Lessons, Book 1* [14] and *Prep Course for Young Beginners: Lesson Book, Level A* [15], these experiences are embedded within short excerpts of music that engage a child’s voice and body percussion [13,16]. The overuse of visual-based methods implicates auditory opportunities in musical development. This results in children not being able to perceive sounds when visual elements of music are present [10], as there is little context for them to tap into to associate [17]. While a child from age three is able to learn symbols and pitch in relation to the piano, there is a lack of auditory-led musical experiences from the onset, let alone being tasked to reproduce the music [6]. According to Sezen [10], visual-based methods should be used to aid the child in perceiving and strengthening the associations among heard sounds. The recommendation to balance the tensions between visual and aural-based approaches is further supported by McPherson and Gabrielsson [6], who recommend that musical notation literacy must be taught in relation to aural perception. According to the authors [6], auditory-led activities should be part and parcel of a piano student’s early musical development. Without providing adequate aural opportunities, we are impeding students from achieving long-term success, as fluency in notation literacy and developing essential musicianship skills hinges on aural abilities.

## 3. Auditory-Teaching Approaches in Piano Pedagogy

Auditory-teaching approaches in music prioritise the person who is learning. They are learner-centred; in early piano instruction, they are child-centred and informed by educational concepts such as child development, the language acquisition process, and experiential learning. As music is an aural art, music educator Shinichi Suzuki believed that early aural exposure to music within a culturally immersed environment allows children to “become natural musicians in the same way that children become natural speakers by hearing language spoken” [17] (p. 47). While listening enables the development of musicianship [17], music educationalists such as Émile Jaques-Dalcroze and Carl Orff believe in engaging the child’s natural facilities to involve the whole body in learning, employing movement and/or voice in varying levels that lead to an aural-led experience with music [4,18,19]. Zoltán Kodály’s holistic approach of incorporating listening, coupled with singing, movement, reading, writing, and improvising, further reinforces the need for aural perceptiveness to develop a melodic sense and the feeling of musical flow [20,21,22]. As Howard [23] pointed out, audiation sensitivity is enhanced through an in-between space, where “alternating between listening and singing” (p. 28) facilitates the translation of sound to symbol. The aural experience of singing develops a strong sense of melodic perception and the inner ear [18]. In Vajda’s [24] guide to Kodály’s approach to learning music, it is evident that the singing voice and some forms of movement are integral and part of his approach. As learning happens through experience, children also learn by inferring music concepts through the engagement of such activities [18]. Auditory teaching approaches in piano pedagogy leverage the same aural-based experiences through rote learning. Young learners can develop coordination and familiarity at the keyboard and make music without the obstacle of notations. In fact, basic aural awareness prior to instrumental tuition could enable learners to progress much faster [25]. The association of sounds and technique is an avenue that allows learners to play songs or pieces that are far more advanced than their actual reading abilities. However, rote learning should not just be mindless imitation; it is the means to developing audiation sensitivity, which fosters an understanding of music [26]. Hence, auditory teaching approaches encourage learners to pause and audiate; in doing so, learners are cultivating their abilities to hear sound mentally (i.e., inner ear). Teacher-guided questioning prompts younger learners to listen with discernment to the elements of music [27].

Research on exploratory listening has mostly focussed on listening, and music creation and reproduction. According to Reybrouck, Podlipniak, and Welch [28], music listening is a complex process in which attention and several processing levels—sensory, physiological, behavioural, and cognitive—are demanded. Reybrouck [29] adds that physical and mental interactions with sounds through listening are acts of musical comprehension, an active process of sense-making. In this article, we explore how auditory approaches in early piano instruction as an epistemic tool may contribute to expanding research on the exploratory behaviour of music listening in young children within the Southeast Asian context of piano learning. Hence, this study seeks to highlight how auditory teaching approaches can be highlighted in early piano instruction as a fundamental tenet in piano pedagogy and how integrating both visual and auditory approaches can balance the tensions created in this in-between space of learning. On this premise, this study addresses the following research question: How do auditory teaching approaches support the understanding of music in early piano instruction?

## 4. Methods

This research is grounded in a multiple case-study methodology to investigate the nuances of learning using both auditory- and visual-based approaches in early piano instruction. Case studies are characteristically used to explore complex, particular phenomena in-depth within a bounded context in time and space [30,31]. This qualitative inquiry is driven by a search for the meaning-making of learning as students navigate between visual and auditory approaches. Three young participants aged 7 and 8 were recruited to examine the teaching approaches in varied sequences or concurrent integration. Two of the three participants were internally recruited from the first author’s private studio, and the remaining participant was recruited externally through an open call. All recruited participants’ learning dispositions and experiences were carefully considered before the assignment of cases to ensure fairness; the participants were in a similar age range for piano learning within early- to mid-beginner proficiency levels and had previous music education experiences.

Prior to undertaking the study, ethical clearance was obtained from the Royal College of Music, London in November 2023, giving assurance to the process of recruitment, confidentiality, and teacher–student–parent relationship in the research. Following a preliminary meeting to provide a detailed explanation of the study with the child and parent, each participant had a total of five sessions spanning three weeks in December 2023. All the materials used in the study were designed by the first author after a review of the related literature [32,33,34]. Except for the semi-structured interview, the parameters of the methods (including lesson plans) were bound to the pedagogical analysis of a prepared piece, *French Child Song, Op. 575 No. 1* by Franz Behr.

**Figure 1:**
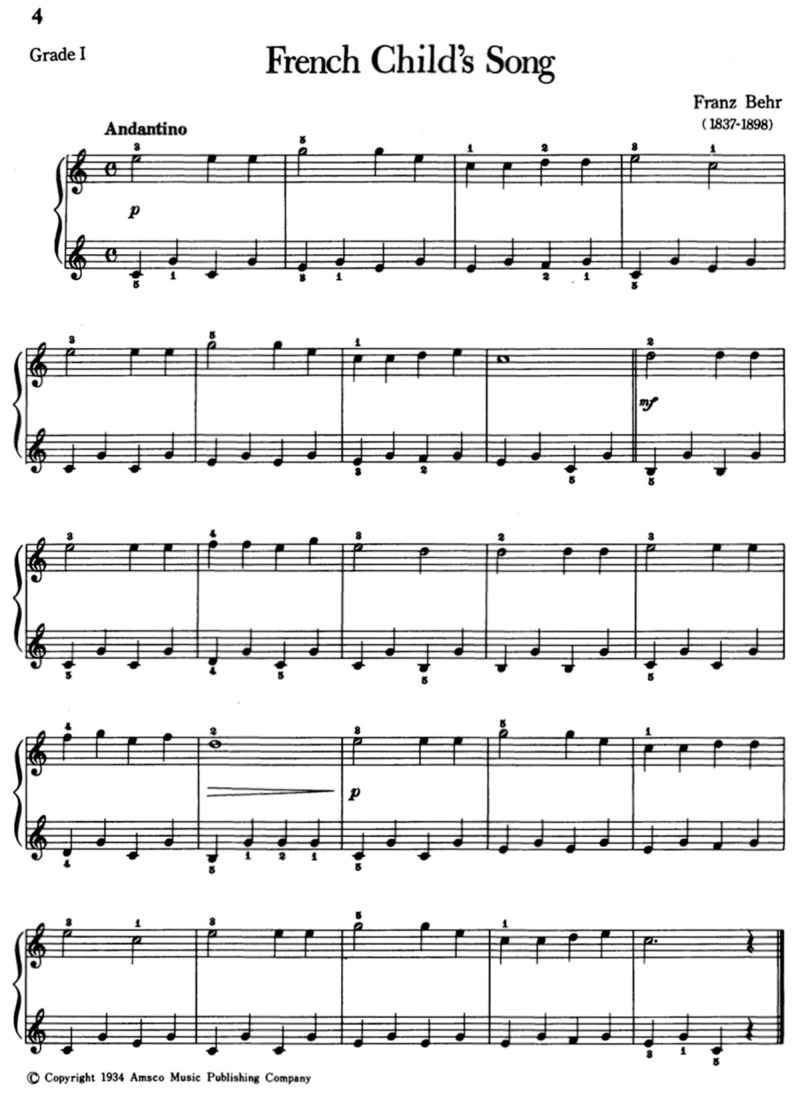
Extract of *French Child Song* by Franz Behr.

The multiple case studies were designed to investigate teaching methods that straddle both visual and auditory approaches in three ways. In Case Study 1, visual-initiated approaches were used before the auditory approach. In Case Study 2, auditory-initiated approaches were used before the visual approach. In Case Study 3, there was a blend in the use of auditory and visual approaches in learning and teaching. Each case was bound to a teaching approach designed to explore the effectiveness of the auditory teaching approaches. While the approaches varied, all participants were given Behr’s work to learn. This piece is composed in C major, 4/4 metre, with simple harmonic progression and ternary form. As shown in Figure 1, the music elements featured were simple and dotted rhythms, with dynamic markings of *p*, *mf*, and *decrescendo* symbols and melodic intervals of 2nds to 6ths. In terms of playing skills, the forearm-led Alberti-like accompaniment is within the penta-scale (i.e., five-finger) position for the right hand, while for the left hand, it extends out, reaching an interval of 6ths at certain points. Using these parameters, the lesson featured modality-flexible activities (e.g., echoing-back with voice or movement or percussive instrument) to work on rhythm that progresses from crotchet to semibreve before teaching dotted minim. Next, rhythm was used as a base to integrate terraced dynamics and gradient inflections before associating the notion of phrasing through contour when the song/piece was sung. The *French Child Song* by Franz Behr was first taught by singing the piece in a solfege system (limited to doh, re, mi, fa, soh, and ti), second, by integrating subtle phrasing and dynamics. All the participants eventually spent time learning technical skills or playing the piece. After each lesson, participants were given homework tasks to complete as part of the uptake in learning and as preparation for the next lesson.

**Figure 2:**
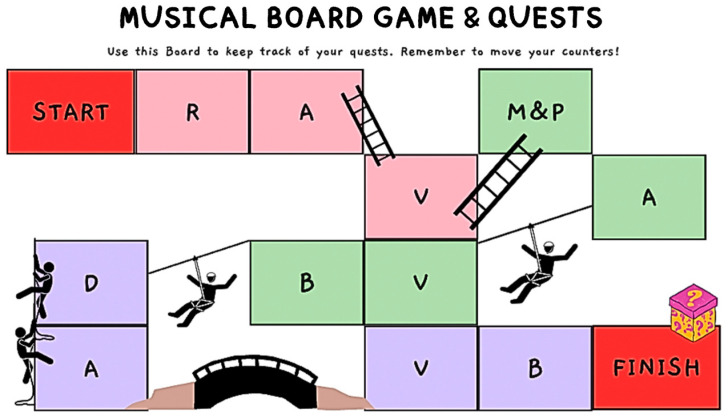
Music board game used in the game-based assessment. The abbreviations indicate the type of music element tested, Rhythm (R), Melody and Phrasing (M & P), and Dynamics (D), and through the type of modality, Auditory (A), Visual (V), and Blended Modality (B). Reprinted from [3].

The data was collected in three stages. In the first stage, the pre-test, game-based assessment (in session 1) was conducted, where participants were assessed through modality-specific (i.e., auditory, visual, and blended) musical games, which examined the learner’s understanding of rhythm, melody and phrasing, and dynamics, based on movement and vocal responses. The materials used in the assessment included a self-designed, step-by-step execution protocol used by the first author, while participants were given a printed board game (see Figure 2). The game included various two-bar rhythmic and melodic patterns, cut-out dynamic symbols, and a whiteboard or A4 notebook. All participants were involved in similar pre- and post-test protocols. In the rhythmic component of the tests, participants were tasked to listen to clapping before responding by clapping back. The melodic components required the participants to listen to ascending and/or descending patterns and skip or step patterns that were played on the piano before being responded to by singing, body gestures, and movements (e.g., walking). The participants were also required to listen to terraced and gradient dynamics in melodic excerpts played on the piano before responding through movement and singing. In addition, participants were required to visually examine several randomised but numbered two-bar excerpts before identifying the notation that resembled what was heard and tasked to draw the contour that expressed the dynamic range.

In the second stage, three lessons (sessions 2–4) that were approximately 45 to 60 minutes in duration were conducted according to the case design. All lessons were taught in two parts: first, musicianship skills, and second, the piano piece, where the teaching was organised by individual music elements (i.e., rhythm, melody and phrasing, and dynamics) or combined together. The instructional approaches toggled between auditory and visual approaches based on the cases assigned. In Case Study 1, visual elements were introduced; this included pre-staff reading, such as pictorial, directional, and solfege-name or letter-name. Rhythmic and dynamic notations, score symbols, and the grand staff were featured as well. Subsequently, in the auditory initiated portions, the musicianship skills and repertoire learnt were reinforced. In Case Study 2, every lesson was predominantly auditory at the start before physical movement, singing, chanting, and playing percussion instruments were introduced. It was only in the last 10 to 15 minutes of the lesson that visual approaches (replicated from Case Study 1) were introduced to reinforce musicianship skills and repertoire. For Case Study 3, the lessons toggled between auditory and visual approaches to reinforce the planned objectives of each lesson. The contents of all lessons were shown through multimedia tools on an iPad.

In the third stage, the evaluation (in session 5) lasted approximately 60 to 90 minutes and included a post-test game-based assessment, a performance assessment, and interviews. The post-test assessment followed the same procedure as the pre-test. The performance assessment of the prepared piece looked at the participants’ understanding of the same three elements and note accuracy on the piano as a medium for musical expression. Lastly, an interview with each participant was conducted to capture insights on teaching design, learning, musicianship skills, and playing abilities. The materials used in the post-test assessment were identical to those used in the pre-test. In the performance assessment, a score was used by the participants to execute a performance rendition of the non-memorised prepared piece. In the interviews, a semi-structured questionnaire was used to guide the teacher and prompt further questions.

The qualitative data of nine recorded lessons (lasting 60 minutes each) were transcribed, thematically analysed, and interpreted by both authors [35]. The process of the initial coding identified four potential themes: recall, association, calibration, and empowerment. The coding process was bound to the interventions (i.e., Case Study 1: Visual to Auditory approaches, Case Study 2: Auditory to Visual approaches, and Case Study 3: blended use of approaches). In the second iteration of coding, the selected incidences were sharpened to generate the defining moments for each theme. The interview data were further triangulated by examining another three recorded post-test, game-based assessments (60 minutes each), and three performance assessments (1 minute each) to fortify the findings on the exploratory behaviours of music listening.

## 5. Findings

Four key themes on the exploratory behaviour of music listening emerged when auditory teaching approaches were integrated with visual teaching approaches: recall, association, calibration, and empowerment (short for R.A.C.E.), the findings addressed the research question of how auditory teaching approaches support the understanding of music in early piano instruction. The findings for Participant 1 described incidences of the visual-initiated teaching approach before the auditory approach. For Participant 2, the incidences described the auditory-initiated approach before the visual approach. For Participant 3, there was a blended use of auditory and visual approaches in learning and teaching. In the following, Participants 1, 2, and 3 will be addressed as P1, P2, and P3.

### 5.1. Recall

Recall, specifically aural recall, requires one to hear and recall patterns of sound through vocal or instrumental means, and it involves a physical production of sound [36]. This provides immediate feedback, enabling the comparison of the previously heard sounds and thus confirming or correcting decisions. There are two types of recall identified from the data: first, the act of assessing the accuracy of decisions with external assistance, and second, self-generated aural feedback. In early music instruction, the former is usually initiated by the teacher, who models by demonstration for learners to respond by imitation [37,38,39].

#### 5.1.1. Externally Assisted Aural Feedback Recall

In Example 1, P1 was tasked to replay a two-bar melodic phrase sung in solfege. This was initially unsuccessful, but after relying on instructor-led aural feedback, the task was completed:

**Example 1.** Participant 1 (Lesson 1).(Section A of the *French Child Song* by Franz Behr was taught before transiting to the auditory approach.) Teacher: Sang mi-mi-mi-soh-soh-mi. P1 responded by playing a short phrase, but suddenly halted halfway. Teacher: (Sang the remaining three notes) Soh-soh-mi. P1’s facial expression changed while straightening the body. P1 played the entire phrase on the piano with the addition of g^5^-g^5^-e^5^.

Externally assisted aural recall was also evident in Example 2. Following through from Example 1, P1 learnt the second section of the piece by reading the notation but unknowingly changed the articulation of a phrase. Instructor-led aural feedback was therefore employed to prompt P1 to listen closely:

**Example 2.** Participant 1 (Lesson 2).(Section B of the *French Child Song* by Franz Behr was taught before transiting to the auditory approach.) Teacher: Sang re-re-re-mi-mi-mi. P1 responded by playing a short phrase with *staccatos* added. Teacher: Listen. Re-re-re-mi-mi-mi. (The teacher sang it with *staccatos* added.) Teacher: Did I do that? Immediately, P1 reattempted to play and rectified the *staccatos* into *legato*.

Similarly, in Example 3, visual approaches were employed before the auditory approaches, with the teacher providing aural feedback to draw P1 to an awareness of a wrong note:

**Example 3.** Participant 1 (Lesson 2).Teacher: Sang fa-soh-mi-soh-mi-re. Was this different from what I have just taught you–what was different? P1 looked around the score for something different. Teacher: Sang fa-fa-mi-soh-mi-re. P1: Fa! Teacher: There are two ‘fa’s. Did you realise that by just listening? P1 replied with a slight nod.

#### 5.1.2. Self-Generated Aural Feedback Recall

In Example 4, aural recall served as a reminder of how music sounded in response to an aural dictation task. In this example, P2 relied on his singing as a reference point to note the solfege and lyrics of the song:

**Example 4.** Participant 2 (Lesson 3).Teacher: Let’s write out the solfege and lyrics of the song. P2: Sang doh-re-mi-doh-re-mi and notates c^4^-d^4^-e^4^-c^4^-d^4^-e^4^ in writing with melodic contour. Teacher: Now write your lyrics. P2 sang the lyrics one two three … three steps up while adding the words.

P2 was also reliant on self-generated aural recall in the game-based test. In this test, the teacher played a melodic pattern starting in *forte* before dropping to *piano* in the last few notes before P2 responded (Example 5):

**Example 5.** Participant 2 (game-based test).Teacher: Played *forte* for c^4^-g^4^-g^4^, followed by *piano* e^4^-d^4^-c^4^. P2 stood up and stomped loudly on the spot before crouching down slightly and lessening the impact as it turned into softer steps. P2: Sang doh-soh-soh-mi-re-doh… It sounds a bit familiar! Teacher: Can you please pick for me which one you have just heard? (Passing the paper with three options for the student to choose.) P2 examined the paper. P2: Doh-soh-soh-mi-re-doh (while softly humming the tune). Number 3! P2 smacked the paper upon identifying the correct notation.

### 5.2. Association

Association is the ability to identify similarities or relate aural stimulus to interdisciplinary ideas and concepts and/or mediums of music–making connections and forming relations. These can vary from identifying similarities to relating heard sounds to interdisciplinary ideas and concepts or other media of music (including using instruments). These associative abilities are observed through children’s kinesthetic, visual, and verbal responses [40,41].

In Example 6, P2 employed movement and drawing media in response to melodic patterns after listening:

**Example 6.** Participant 2 (Lesson 3).Teacher: Based on the pitch changes, if I move the pitch up or down, you will respond by moving your body up or down. Teacher: Plays: c^5^-d^5^-e^5^-g^5^. P2 responded by moving from a crouched position to standing. Teacher: Very good. Now change your position accordingly to this tune. Teacher: Played e^4^-f^4^-g^4^-f^4^-e^4^-d^4^-c^4^. P2 showed the changing pitches through movement while humming the tune. P2: Sang mi-fa-soh-fa-mi-re-doh. Teacher: Okay. If I would ask you to draw this–how would you draw it? P2 proceeded to show the melodic contour by drawing on the whiteboard. A swift freehand melodic contour was drawn first… P2 then re-attempted to redraw and added bar lines. 
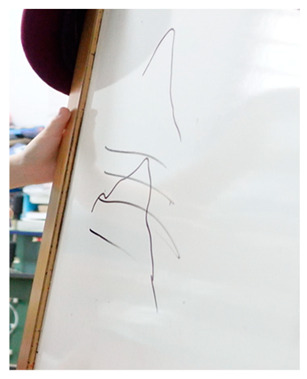


Similarly in Example 7, P1 responded to an aural pattern by expressing it through a visual drawing of the melodic contour:

**Example 7.** Participant 1 (Lesson 3).Teacher: Let’s draw out the melody we hear. (Played c^4^-e^4^-d^4^-f^4^.) Teacher: Is yours similar to mine? P1 (left) and Teacher (right) compared answers by tracing the contour. 
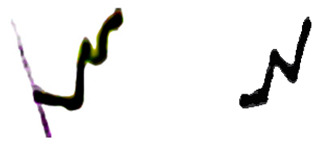


In Example 8, P3 responded to a melodic pattern that sounded familiar and expressed it through movement in the blended test of both auditory and visual approaches that was administered:

**Example 8.** Participant 3 (game-based test).Teacher: Plays e^5^-e^4^-g^5^-e^5^-e^5^. Do you think the melody was going up or down- P3 (before the teacher could finish): Why does this melody feel like it is inside there (pointing to the prepared piece taught in the lesson)? Teacher: Do you think the melody was going up or down? P3: Going up! (At this moment, P3 reached out and grabbed two wooden blocks. P3 processed and elaborated on the heard sounds by showing them visually through embodied movements.) P3: Going up like this. (P3 demonstrated by stacking two blocks on top of each other.)

### 5.3. Calibration

Recalling through calibration is often employed as trial and error during the learning and refinements of performance work, or by play-by-ear using the piano [36]. When learners employ recalling from stored memory, it allows the recognition of errors in their playing [42], enabling the monitoring of their own playing standards by comparing it to the previously heard model during instruction. Calibration involves the same act of assessing the accuracy of decisions; however, it involves recalling sounds from memory by tapping into the stored memories of sound that serve as a reference point, enabling the comparison of the previously heard sounds, thus confirming or correcting their decisions.

#### 5.3.1. Calibration in Learning Activities

In Example 9, the teacher provided P1 with verbal feedback to draw awareness to a wrong note:

**Example 9.** Participant 1 (Lesson 1).(Section B of the *French Child Song* by Franz Behr was taught before transiting to the auditory approach. P1 responded by playing a short phrase on the piano after listening to the teacher’s singing; however, P1 halted halfway through.) Teacher: Sang doh-doh-re-mi-doh. P1: Plays c^5^-c^5^-d^5^-d^5^-e^5^-d^5^. Teacher: Was there a wrong note? P1 immediately paused to think and rectified the phrase c^5^-c^5^-d^5^-e^5^-c^5^.

In Example 10, P2 demonstrated calibration of what was heard internally during the process of adding dynamic symbols (i.e., *crescendo* and *decrescendo*) to the score:

**Example 10.** Participant 2 (Lesson 2).Teacher: Now echo after me. Mi-mi-mi-soh-soh-mi. Teacher: Did you hear that I have added the shaping—the dynamics? P2: Yes. P2 proceeded to play e^4^-e^4^-e^4^-g^4^-g^4^-e^4^ with dynamic shaping on the xylophone. The score of the *French Child Song* was shown to the student. Teacher: Now we move to the visual part of the activity. P2: Can we at least draw out *crescendo* and *decrescendo*? Teacher: Of course. … Can we add in the shaping now instead, and even with phrasing? P2 proceeded to determine the dynamic shaping by adding it to the score.

In Example 11, P3 was tasked to echo a short passage of Alberti bass through the xylophone but struggled to identify one of the missing notes. This resulted in the teacher switching to visual aids while singing the solfege, triggering a response whereby P3 recalled the Alberti pattern:

**Example 11.** Participant 3 (Lesson 2).Teacher: Sang ti-soh-ti-soh-doh-soh-doh-soh. P3 proceeded to echo the phrase on the xylophone. Teacher: But where is your doh note? P3 started to show signs of confusion. The teacher intervened by providing visual cues while singing and pointing at the notation. Teacher: Sang ti-soh-ti-soh-do-soh-do-soh. P3: Why do I find it familiar? Teacher: This is your left-hand pattern. P3: Oh, that’s right!

#### 5.3.2. Calibration During Performance Test

Cognitive demands were most apparent during the assessed performance tests, where visual processing hindered fluency. In the following three examples (Examples 12–14), P1, 2, and 3 displayed challenges processing the notation but subsequently calibrated their actions through active aural detection:

**Example 12.** Participant 1 (Performance Test).P1 performed the *French Child Song* with the score on the piano. In the repeat of Section A, P1 paused after a few notes into the bar and realised that the tune did not sound right. P1 leaned forward to check and restarted the phrase, successfully changing the wrong note from c^5^ to e^5^.

**Example 13.** Participant 2 (Performance Test).P2 performed the *French Child Song* with the score on the piano. During the transition to Section B, P2 paused after playing a wrong note and realised that the tune did not sound right. P2 quickly checked for the right note and restarted from where he had stopped.

**Example 14.** Participant 3 (Performance Test).P3 performed the *French Child Song* with the score on the piano. During the transition to Section B, P3 paused, as it was difficult to read and play fluently on time. P3 leaned forward trying to decode what the next note was. After recognising the note, P3 continued playing but almost immediately played a wrong note. P3: Tsk! (P3 reacted in displeasure, realising that the sound did not match what was expected from its tune, but proceeded to retry.)

### 5.4. Empowerment

Empowerment highlights incidences of learners’ musical agency, where actions are a display of “desire and capacity to initiate and carry out their musical ideas” [43] (p. 115). Auditory-teaching approaches empower learners to be in a state filled with autonomy and agency, and to take ownership of the task with a certain level of confidence as a result of aural familiarity.

As shown in Example 15, P2 was able to draw from the auditory approaches that were experienced at the start of the lesson and execute the rhythm without assistance:

**Example 15.** Participant 2 (Lesson 1).Auditory approaches were employed from the start of the lesson in rhythmic activities before this visual activity was employed near the end of the lesson. Teacher (Providing the visual illustration): Could you be player 2, and I will be player 1? Please also select an instrument of your choice. P2: Picked up the castanet and proceeded to execute the rhythmic notation independently.

In Example 16, empowerment was also observed after auditory teaching approaches were used to circumvent P2’s apparent obstacles:

**Example 16.** Participant 2 (Lesson 3).P2: I am always struggling with this part (while pointing at the score). P2 proceeded to demonstrate the passage that was problematic. Teacher employed chunking strategy with altered rhythm and sang the solfege of melody and accompaniment separately. Teacher: Now, your left hand will play soh-doh, and your right hand play fa-mi (while covering the score that was in front of P2). P2: Played both hands separately as instructed before combining both hands together. Teacher: Okay, next. (This was repeated for several other bars.) Teacher: Now, starting from Section B (while pointing at the score). (The focus was redirected back to the visual approach.) P2: Executed both hands without any problems. Teacher: Is it easier now? P2: Yes.

## 6. Discussion

This study sheds light on fresh approaches for discussing how we may uncover nuanced differences in learning between auditory and visual teaching approaches. In Case Study 1, the shift from visual to auditory approaches gave rise to both instructor-led and learner-generated aural recalls. As shown in Example 1, P1 had initially learnt the prepared piece through a visually illustrated version of the score. However, during an echo-back activity where the teacher sang a short melody from the music, P1 could not fully execute it back on the piano. This prompted instructor-led aural recall to help P1 make progress. Similarly, in Example 2, the teacher employed instructor-led aural recall to point out mistakes in articulation, while in Example 3, the teacher deliberately sang an incorrect note to prompt the student to make a comparison. Such incidents of instructor-led aural recall became a tool for ‘teachable’ moments. In this process, through music listening, the learners’ association of heard sounds was strengthened. While Kerchner [40] suggested that children are able to engage in aural recall by singing back a tune to illustrate their perceptions of heard music, this study observed that instructor-led aural recalls are equally important in music learning. This finding corresponded with Wickes [42], who highlighted the fact that a teacher’s aural or physical demonstration serves as a model for learners to benchmark and compare their efforts. This affirms why aural recall through vocal or instrumental means is seen as one of the many musicianship skills that are important for and expected of music learners.

In Case Study 2, the shift from auditory to visual approaches empowered the participant with agency and the ability to complete tasks. The aural-centred activities experienced in the lessons (as cited in Examples 5, 6, and 10), including active musical play through vocalisation, rhythmic bodily movements or use of rhythmic objects, and playing instruments [44], provided crucial aural familiarity which consequently empowered P2 to pick up the castanet and independently execute the rhythmic pattern notated on a score, as evinced in Example 15. This corresponded with Wiggins [43] on how musical agency is grounded and unfolds “from knowledge constructed through prior musical experience—including musical enculturation, informal music learning, and formal music learning” (p. 115). As an intervention strategy, auditory approaches facilitated P2 to divert obstacles and encouraged attention to listen and coordinate on performance-based tasks. This finding highlights how auditory approaches aid in assisting attention during visual score reading by reducing the overload of musical information, allowing individuals to concentrate and tune in to specific performance-based tasks in learning. As shown in Example 16, P2 clearly expressed his struggles at points where patterned changes occurred in either hands or during system changes. This led to the teacher removing the visual obstacle (i.e., notation) and diverting attention to the hands and ears through the auditory instruction to work on the coordination of hands.

In Case Study 3, the immediate switch between aural and visual approaches in Example 11 showed how calibration was enabled, corresponding with Dunn’s [41] affirmation that children are able to demonstrate the ability to make comparative statements between what was previously heard and what is being heard during the current session. The switches also enabled an aural association to occur for P3, as shown in Example 8, in which the author [41] also mentioned that children were able to demonstrate abilities of kinesthetic and visual association through movements that depicted heard sounds.

Interestingly, in all of the case studies, calibrative behaviours were observed frequently for all participants. Prevalent in the performance assessments (as cited in Examples 12—14), the data showed how all three participants were able to monitor their playing through listening––consequently making the appropriate amendments. This finding concurs with Hallam [45], who suggested that such monitoring ability in performance is possible after the acquisition of an aural template––that is, the internalisation of sounds. Through listening and appraising, learners can detect errors by comparing them to their internal representations as reference points, affirming that aural familiarity does play a part in children’s error detection abilities [46].

As the case studies demonstrate clearly, the act of switching between auditory and visual approaches was an invaluable experience that enabled the participants to appreciate a multitude of ever-shifting perspectives in learning. The findings further unpack how young learners have experienced learning in the space in-between. There was an element of demonstrable discomfort for all participants. For P1 (in Example 3), moments of uncertainty were observed in the participant’s search for an answer. In the cases of P2 (in Example 10), and P3 (in Example 11), these participants somewhat struggled when they had to switch from aural to visual expressions of learning. P2 was even able to clearly express his struggles in shifting from an auditory understanding to a visual interpretation of notations.

Such states of disempowerment are not unusual as learners shift modalities of learning. What is, however, useful to the teacher is to acknowledge these emotional states of learning as a journey that will need to be continually refined by an in-between space so that learners can begin to take stock of such uncomfortable experiences in order to find new ways of toggling between visual and aural literacies to find new ways of integrating musical perspectives. As individuals dwell in a state of peripherality [47], they begin to legitimise a peripherality that adds value to learning [5].

As in the aforementioned cases, in which the findings show learners performing higher levels of cognitive function, we recognise the cognitive demands in music learning through music listening during the execution of musical tasks and music-making, as cited in instances of association and calibration in Examples 6, 8, and 10. These incidences also shed light on how essential musicianship abilities are sowed and nurtured. This study observed the development of musical comprehension and performance-based skills happening in the process of learning. In addition, the findings further highlight the demanding cognitive ability needed to process visual elements in score reading. We believe that these findings will be of interest to readers, demonstrating auditory approaches can complement attention given to visual score-reading, enabling students to better tune in to the coordination of their hands with the music.

## 7. Conclusions: A R.A.C.E. Toward an In-Between Space of Learning

Auditory teaching approaches catalyse to build listening acuity and acuteness in learners, as observed through the incidences of recall, association, calibration, and empowerment (R.A.C.E.). As the findings have shown, both active and reactive listening were central to learners’ navigation and interaction with sounds. While recall, association, and calibration can be conceived of as separate abilities, they form the essential mechanisms in which learners comprehend music in learning and performing.

The authors are aware that, when it comes to pedagogical approaches, there is no one best way. Hence, we concern ourselves by designing teaching methods that straddle in-between approaches. The data revealed R.A.C.E. as the positive instances of an in-between space of learning in exploratory behaviours for music listening. Having focussed on listening and music learning, the emphasis on auditory teaching approaches allowed young learners to be active participants in their music learning, during which they hear, listen, sing, move, and explore through music. Through this integration, we impact learners’ experiences on several levels as we make learning holistic and musically concrete. While this study presented findings on how auditory teaching approaches (in its various forms) support the understanding of music in early piano instruction, the study is limited by a homogeneous composition of three male Chinese participants (7 and 8 years old). Due to the smaller-than-usual sample size of participants in this pilot study, the findings uncovered may not be broadly generalised or represented in all teaching contexts. However, these findings remain valuable insights that were corroborated through data triangulation. Additionally, all participants had dissimilar prior learning experiences. Specifically, Participants 1 and 2 had received prior training from the first author for 2 years, while Participant 3 was externally recruited through an open call. While Participant 3 acclimated very well, the authors are aware that this limits the study’s findings due to differentiated familiarity in both teaching styles and musical backgrounds. Future research should ensure the recruitment of a well-represented population of young learners—a heterogeneous group of gender, ethnicity, music, and piano backgrounds. Lastly, future research should consider scaling up for a longitudinal study to uncover a wider population of young learners and variously applied instrumental teaching settings. As the study was conducted in the participants’ homes, the first author was unable to control the learning environment. While this is reflective of realistic teaching practices, future research should consider a designed environment to observe teacher-student interactions. Nonetheless, this study was able to standardise conducive learning conditions for all participants. The focus of the study was on the effectiveness of auditory teaching approaches in response to the gap in early piano instruction to improve musical comprehension in children. Future possibilities may go beyond validating and may expand into related domains of musicianship. For example, one could examine auditory teaching approaches in relation to music notation literacy. This could deepen the understanding of music listening and music learning, as developing the musician–pianist identity is paramount; the essential musicianship skills that form the basis for future development in one’s musical life.

## Data Availability

The datasets presented in this article are not readily available because of ethical reasons. Requests to access the datasets should be directed to the Institutional Review Board (or Ethics Committee) of the Royal College of Music, London.
